# Insights Into the Genetic Diversity of Nordic Red Clover (*Trifolium pratense*) Revealed by SeqSNP-Based Genic Markers

**DOI:** 10.3389/fpls.2021.748750

**Published:** 2021-10-25

**Authors:** Johanna Osterman, Cecilia Hammenhag, Rodomiro Ortiz, Mulatu Geleta

**Affiliations:** Department of Plant Breeding, Swedish University of Agricultural Sciences, Lomma, Sweden

**Keywords:** DAPC, gene targeting markers, heterozygosity, loci under selection, population structure, red clover, SeqSNP

## Abstract

Red clover (*Trifolium pratense*) is one of the most important fodder crops worldwide. The knowledge of genetic diversity among red clover populations, however, is under development. This study provides insights into its genetic diversity, using single nucleotide polymorphism (SNP) markers to define population structure in wild and cultivated red clover. Twenty-nine accessions representing the genetic resources available at NordGen (the Nordic gene bank) and Lantmännen (a Swedish agricultural company with a red clover breeding program) were used for this study. Genotyping was performed via SeqSNP, a targeted genotype by sequencing method that offers the capability to target specific SNP loci and enables *de novo* discovery of new SNPs. The SNPs were identified through a SNP mining approach based on coding sequences of red clover genes known for their involvement in development and stress responses. After filtering the genotypic data using various criteria, 623 bi-allelic SNPs, including 327 originally targeted and 296 *de novo* discovered SNPs were used for population genetics analyses. Seventy-one of the SNP loci were under selection considering both Hardy-Weinberg equilibrium and pairwise F_ST_ distributions. The average observed heterozygosity (H_*O*_), within population diversity (H_*S*_) and overall diversity (H_*T*_) were 0.22, 0.21 and 0.22, respectively. The tetraploids had higher average H_*O*_ (0.35) than diploids (0.21). The analysis of molecular variance (AMOVA) showed low but significant variation among accessions (5.4%; *P* < 0.001), and among diploids and tetraploids (1.08%; *P* = 0.02). This study revealed a low mean inbreeding coefficient (F_IS_ = −0.04) exhibiting the strict outcrossing nature of red clover. As per cluster, principal coordinate and discriminant analyses, most wild populations were grouped together and were clearly differentiated from the cultivated types. The cultivated types of red clover had a similar level of genetic diversity, suggesting that modern red clover breeding programs did not negatively affect genetic diversity or population structure. Hence, the breeding material used by Lantmännen represents the major genetic resources in Scandinavia. This knowledge of how different types of red clover accessions relate to each other and the level of outcrossing and heterozygosity will be useful for future red clover breeding.

## Introduction

Red clover (*Trifolium pratense*) is an important crop to secure sustainable cattle farming and thus meat and dairy production, as it is one of the most important forage legumes worldwide (Smith et al., 1985; Taylor and Quesenberry, 1996a). It is a perennial forage legume, which can be harvested multiple times within a year. Red clover is favored due to its nutritional value and positive effect on soil quality. It grows in symbiosis with nitrogen fixing bacteria in the rhizosphere, thus increasing soil fertility (Sturz et al., 1997; Thilakarathna et al., 2017). Until the 1930s, when industrial fertilizers started to be widely used, red clover was an important crop for the European agriculture mainly due to its symbiosis with rhizobacteria, thereby securing high yielding harvests (Kjærgaard, 2003).

According to Merkenshlager (1934), red clover was first cultivated in Spain during the 13^*th*^ century and then spread throughout Europe. Records show that it has been cultivated in the southern parts since the 1560s and in the northern parts since the 1770s. It continued to spread to the temperate regions of North and South America, to New Zealand and Australia and, to China and Japan (Taylor and Quesenberry, 1996a). Red clover is naturally diploid (2n = 2x = 14; Evans, 1954), which includes wild populations as well as landraces and traditional cultivars. However, modern autotetraploid red clover cultivars (2n = 4x = 28) have been developed from diploid genotypes through chromosome doubling (Sjödin and Ellerström, 1986; Taylor and Quesenberry, 1996b).

The financial sustainability of small-scale farmers, in competition with bigger foreign markets, is an important aspect to be considered with regard to the production of local and sustainable beef and dairy products, as already noted in Sweden (Fischer and Röös, 2018). Even though the farmers in Sweden follow quality-grazing requirements, the cattle still require additional fodder to ensure their high quality and reliable milk production as well as the health and development of their calves (Åkerlund, 2008; Kilsgård, 2015). The requirement of adding, for example, soybean meal to the fodder to increase crude protein levels is not sustainable for cattle farming in northern Europe or elsewhere where there is no soybean production. The development of local red clover cultivars are therefore important for the global beef and dairy industry.

To assist farmers in providing good quality beef and dairy to consumers, plant breeders need to develop red clover cultivars that have great nutritional value, along with superior yield. Additionally, the crop should show high persistence under local climates and biotic stresses. For example, the root-rot disease caused by fungal pathogens can wipe out an entire field of red clover in the absence of pesticide use (Bengtsson, 1961). The fungicide used to prevent root-rot in the late 1900s is not approved by environmental agencies (United States Environmental Protection Agency, 2006). Hence, the best way to prevent the disease is to develop resistant cultivars against the pathogen. Persistence, under biotic and abiotic stresses, is a difficult trait to breed for and so far, only one quantitative trait locus (QTL) linked to the persistence trait has been detected in *T. pratense* (Herrmann et al., 2008). Unique climate zones, such as the lands closer to the polar circle in the northern hemisphere can be highly variable in temperature, precipitation and day length due to seasonal changes. Thus, there are additional requirements to realize persistent red clover cultivars suitable for mid and high-latitude regions.

DNA markers have been widely used to conduct population genetics research in crops and their wild and weedy relatives, both for understanding their genetic makeup as well as to establish their genetic and phylogenetic relationships. SeqSNP is a targeted genotype by sequencing method that can be designed for genotyping of known highly polymorphic SNPs. If desirable SNPs are unavailable, novel SNP candidates can be identified through allele mining of genomic resources of a target crop from the GenBank. The significance of these novel SNPs can be evaluated by using them for population genetic studies. Based on the results, a core set of SNPs can be developed for various applications including marker-aided breeding. *T. pratense* is among the crops with publicly available whole genome sequences (GenBank accessions: GCA_000583005.2, GCA_900079335.1 and GCA_900292005.1) (Istvánek et al., 2014; De Vega et al., 2015). The latest one, GCA_900292005.1, is a chromosome level assembly with a total sequence length of 351.6 Mbp.^1^ This assembly shows high synteny between *T. pratense* and *Medicago truncatula*, a model species for the legume family (Cook, 1999; Frugoli and Harris, 2001). A coding sequence assembly based on a cultivated red clover population is also available at the Legume Information System (LIS^2^). The annotation of the assemblies can be used to locate gene sequences and thus identify SNPs targeting desired genes.

As explained by Poczai et al. (2013), gene targeting markers (GTMs) is a category of markers that target specific genes. When located within the coding regions of a specific gene, GTMs differ from non-genic markers that are more reliant on association with target genes or loci. By developing SNPs within coding regions of a gene, the likelihood of accurately describing potential genotypic variation with regard to a specific trait increases. This enables the description of genetic diversity within and among populations with respect to important traits (van Tienderen et al., 2002). The aim of the present study was to use SNPs as GTMs to determine the genetic diversity and population structure of red clover represented by its genetic resources available in Northern Europe, where it is a major forage legume.

## Materials and Methods

### Plant Material and Sampling

Twenty-nine red clover accessions were used for this study (Table 1). Twenty-one of these accessions were obtained from NordGen, a genebank for genetic resources in the Nordic countries, by ordering via Nordic Baltic Genebanks Information System (GeNBIS),^3^ whereas the remaining eight were obtained from Lantmännen Seed,^4^ a plant breeding and agricultural seed company based in Sweden. The NordGen accessions were selected based on their passport data and represent cultivars, landraces and wild forms from the Nordic countries, as well as one Russian accession collected near the Finnish border. The accessions from Lantmännen represent cultivars and synthetic populations and include both diploids and tetraploids. Hereafter, cultivars from NordGen and Lantmännen will be referred for distinction as cultivar_*N*_ and cultivar_*L*_, respectively.

**TABLE 1:** Summary of the accessions with origin, type (B = breeding population, C_*L*_ = cultivar from Lantmännen, C_*N*_ cultivar from NordGen, L = landrace population, S = synthetic population, W = wild population), percent polymorphic loci (%PL), within population diversity (H_*S*_), observed heterozygosity (H_*O*_), inbreeding coefficient (F_IS_), discriminant analysis of principal components (DAPC) cluster designation and Nei’s standard genetic distance (NSGD).

Accession	Origin	Type	%PL	H_*S*_	H_*O*_	F_IS_	DAPC clusters	NSGD	
								Mean	SD
NGB1730 ^c^	Denmark	C_N_	65.0	0.21	0.20	0.01	1 and 2; 70%, 30%	0.026	0.005
NGB1743 ^c^	Denmark	C_N_	67.1	0.21	0.20	0.02	1 and 2; 60%, 40%	0.026	0.006
NGB4126 ^c^	Denmark	C_N_	63.9	0.19	0.19	0.00	1 and 2; 90%, 10%	0.025	0.005
NGB8393 ^c^	Denmark	C_N_	64.5	0.21	0.20	0.01	1 and 2; 80%, 20%	0.024	0.005
NGB9228 ^c^	Denmark	C_N_	65.7	0.21	0.21	0.01	1 and 2; 60%, 40%	0.027	0.005
NGB11586 ^c^	Denmark	C_N_	61.0	0.21	0.19	0.00	1 and 2; 75%, 25%	0.029	0.006
NGB11605 ^c^	Denmark	C_N_	58.1	0.20	0.22	–0.08	1 and 2; 50% each	0.025	0.005
NGB11608 ^c^	Denmark	B	58.4	0.19	0.19	0.02	1 and 2; 20%, 80%	0.025	0.005
NGB1142 ^c^	Finland	L	70.3	0.22	0.21	0.03	1 and 2; 50% each	0.025	0.005
NGB14327 ^c^	Finland	L	68.2	0.21	0.21	–0.01	1 and 2; 70%, 30%	0.026	0.005
NGB14444 ^c^	Finland	C_N_	63.6	0.19	0.20	–0.03	1 and 2; 80%, 20%	0.026	0.005
NGB14448 ^c^	Finland	W	61.3	0.21	0.21	–0.03	1 and 2; 50% each	0.024	0.005
NGB2194 ^c^	Norway	L	64.7	0.20	0.20	–0.02	1 and 2; 90%, 10%	0.026	0.005
NGB2202 ^c^	Norway	L	63.7	0.20	0.19	0.05	1 and 2; 60%, 40%	0.027	0.006
NGB15558 ^c^	Norway	W	53.1	0.18	0.19	–0.06	3	0.025	0.005
NGB15623 ^c^	Norway	W	59.4	0.19	0.19	–0.02	1 and 2; 50% each	0.026	0.005
NGB1009 ^c^	Sweden	W	48.2	0.16	0.16	0.01	3	0.026	0.005
NGB1420 ^c^	Sweden	W	55.7	0.18	0.18	0.01	3	0.024	0.005
NGB6184 ^c^	Sweden	L	70.3	0.21	0.20	0.05	1 and 2; 60%, 40%	0.026	0.005
NGB9966 ^c^	Sweden	L	65.8	0.21	0.19	0.06	1 and 2; 50% each	0.027	0.006
NGB24176 ^c^	Russia	W	54.4	0.16	0.16	0.00	3	0.024	0.005
LÖRK0390 ^a^	Lantmännen	S	68.7	0.22	0.22	–0.02	1 and 2; 50% each	0.026	0.006
SW ARES ^a^	Lantmännen	C_L_	64.5	0.21	0.21	–0.05	1 and 2; 70%, 30%	0.024	0.005
SW YNGVE ^a^	Lantmännen	C_L_	65.0	0.21	0.19	0.03	1 and 2; 30%, 70%	0.025	0.005
SWÅ RK07001 ^a^	Lantmännen	S	65.7	0.21	0.21	0.01	1 and 2; 50% each	0.026	0.005
SWA 1675209 ^b^	Lantmännen	S	71.3	0.25	0.34	–0.31	1 and 2; 10%, 90%	0.024	0.005
SW RK1158 ^b^	Lantmännen	S	73.8	0.25	0.36	–0.33	2	0.024	0.005
SW RK1166 ^b^	Lantmännen	S	75.9	0.26	0.35	–0.29	1 and 2; 40%, 60%	0.026	0.006
Vicky ^b^	Lantmännen	C_L_	73.8	0.25	0.34	–0.29	1 and 2; 40%, 60%	0.025	0.005
Mean			64.2	0.21	0.22	–0.04		0.025	0.005

*^*a*^ = known diploid; ^*b*^ = known tetraploid; ^*c*^ = ploidy not determined.*

A minimum of 20 seeds were planted for each accession in a greenhouse at the Swedish University of Agricultural Sciences (SLU, Alnarp, Sweden) in May 2020. For each accession, two 2 L plastic pots filled with soil were used for planting. Poorly germinating seeds were treated by manual scarification according to Asci et al. (2011) and placed on Petri dishes until germination was achieved, and then transferred to pots. Two weeks after planting, extra seedlings were removed and five seedlings per pot were maintained. Later, leaf tissue from ten individual seedlings per accession were separately sampled, except for accession NGB11586, which was represented by eight individual seedlings. Hence, 288 individuals originating from different seeds were separately sampled in total. For sampling the leaf tissue, BioArk Leaf collection kit provided by LGC, Biosearch Technologies^5^ was used. From each plant, ten 6 mm leaf discs were sampled using a punch and were put in a sampling plate. The collected leaf tissue was then sent to LGC, Biosearch Technologies (Berlin, Germany) for DNA extraction and subsequent genotyping. Using the Sbeadex plant kit,^6^ high quality genomic DNA was extracted for SeqSNP genotyping.

### Single Nucleotide Polymorphism Selection, SeqSNP Assay Design and Sequencing

A literature review was performed on red clover targeting pathways of growth and development as well as stress and disease responses. Based on this foundation, genes related to disease resistance, stress response (such as hormone regulation or interaction) or growth and development were targeted for single nucleotide polymorphism (SNP) mining. The SNP mining was performed using coding sequences (CDS) of red clover genes downloaded from the legume information system (LIS) database^2^ where the CDS of genes of interest were aligned against the red clover reference genome [*Trifolium pratense* genome v3, GenBank assembly (GCA_900292005.1)] using the Basic Local Alignment Search Tool (BLAST) of the National Center for Biotechnology Information (NCBI). As only CDS sequences with a single hit in the red clover genome were used for the SNP identification, all SNPs used in this study were considered to be from single-copy genes. If a CDS could not be clearly associated with the list by its associated protein name, the UniProt database^7^ was used to identify the related pathway. If the related pathway was in accordance with the selection criteria, the sequence was chosen for the SNP mining. Alignments were accepted at a threshold of 95% sequence identity between the query and subject sequences. Whenever more than one SNP was selected per target sequence, the SNPs were at least 55 bp apart. In total, 641 target SNPs in 324 CDS sequences were used for SeqSNP assay design. Of the 641 target SNPs, 571 SNPs were fully covered (two oligo probes per target) and passed high specificity assay design (no off-target hits allowed), whereas the remaining 70 SNPs failed. Among the 571 SNPs that passed the high specificity assay design, 400 target SNPs in 247 sequences were then selected for SeqSNP analysis by taking into account the distribution of the SNPs across the genome, the function of the genes as well as the distance between SNPs within a gene (Supplementary Table 1). In this final set, the SNPs within a given sequence were at least 70 bp apart. Then, SeqSNP kit containing 800 high-specificity oligo probes for the 400 SNPs were produced, a sequencing library was constructed and the target SNPs were sequenced. The Illumina NextSeq 500/550 v2 platform was used for sequencing in 150 base-pair (bp) single read mode. On average, ca 217,000 reads per sample was conducted, and the average effective target SNP coverage was 501x.

### Variant Discovery, Genotype Assignment, and Data Filtering

After sequencing, the raw reads were adapter-clipped and quality-trimmed (reads containing Ns removed, reads trimmed to obtain a minimum average Phred quality score of 30 over a window of ten bases, and reads with final length < 130 bp discarded). For genotype calling, the SNP genotyping pipeline was set to diploid genotyping with a minimum coverage of eight reads per sample per locus, as described below. The alignment of quality trimmed reads against the reference genome using Bowtie2 v2.2.3 (Langmead and Salzberg, 2012) and variant discovery and genotyping of the samples using Freebayes v1.0.2-16 (Garrison and Marth, 2012) showed that out of the 400 target SNP loci, 15 were monomorphic across the 288 individuals. Furthermore, among the 385 polymorphic loci, 352, 30 and 3 were bi-, tri- and tetra-allelic, respectively, within the genotyped samples. Mono-, tri- and tetra- allelic SNP loci were excluded from further analysis. For genotype determination of the bi-allelic loci, alleles with an allele-count of less than eight were set to missing, according to the threshold set for the genotype-calling pipeline, as a procedure to exclude alleles called due to a potential sequencing error. Although the ploidy level of the NordGen accessions were not indicated in their passport data, the wild populations, landraces and traditional cultivars are diploids, as described in the “Introduction.” The accessions labeled as cultivar_*N*_ were also considered diploids based on observed phenotypic characteristics. On the other hand, the four accessions from Lantmännen are known tetraploids. A preliminary genetic diversity analyses of these tetraploid accessions conducted using their allele frequency data, calculated from their allele counts, provided similar results with the results obtained by treating them as diploids. Hence, the genotype of each sample at each locus was determined based on the allele-count, as diploid for all accessions in the final data analyses. Hence, individuals having only one allele with an allele-count of above eight were recorded as homozygotes whereas those with an allele-count of above eight for two alleles were scored as heterozygotes. The bi-allelic SNP data were then filtered to retain only loci with missing data of less than 5%. Among the 352 bi-allelic SNPs, 21 had a missing data of over 5%, thus only 331 were retained for further analysis. In addition to the target SNPs, 292 bi-allelic SNPs that fulfilled all aforementioned filtering criteria were identified *de novo* within 75 bp range on both sides of the target SNPs.

### Analysis of Molecular Variance, Population Structure and Cluster Analyses

R (R Core Team, 2013) was used to assess whether the SNPs were in agreement with the assumptions of an outcrossing crop by computing expected and observed heterozygosity and F-statistics. The analysis was performed using the method proposed by Kamvar et al. (2017) using the R package adegenet version 2.0.0 (Jombart, 2008) and hierfstat version 0.5–7 (Goudet, 2005). The analysis of molecular variance (AMOVA) was performed using Arlequin suite version 3.5.2.2 (Excoffier and Lischer, 2010) without grouping the 29 accessions as well as by grouping them based on different criteria, as presented in the results. To determine the extent of differentiation between the populations, the F_ST_ (fixation index; Wright, 1931) and Nei unbiased genetic distance (Nei, 1987) between subpopulations were calculated using Arelquin ver 3.5.2.2. The principal coordinate analysis (PCoA) was calculated in R with the stats package (R Core Team, 2013) based on the Nei unbiased genetic distance matrix. The neighbor-joining (NJ) tree at a population level was constructed using MEGA7 (Kumar et al., 2016) based on the Nei unbiased genetic distance matrix. Additionally, a NJ tree at individual sample level was constructed using MEGA7 based on a pairwise genetic distance matrix calculated using the Tamura and Nei (1993) method. The discriminant analysis of principal components (DAPC) was performed in R using the adegenet package on individual genotypic data and 100 principal components based on the method of Jombart et al. (2010). The principal components were selected using a function from the adegenet package that evaluated the most informative principal components using cross-validation.

### Hardy-Weinberg Equilibrium and Neutrality Tests, and Mutation Analysis

Arlequin suite version 3.5.2.2 was used to identify loci that significantly deviated from the Hardy-Weinberg equilibrium (HWE) assumptions, at 0.05 level of significance. For this analysis, 1000,000 steps in Markov-chain and 100,000 dememorization steps were used. Similarly, Arlequin was used to detect loci under selection (at 0.05 level of significance) through the examination of the joint distribution of F_ST_ and heterozygosity under a non-hierarchical finite island model, using 20,000 coalescent simulations (Excoffier et al., 2009). This led to the identification of 51 loci that deviated from HWE (each with a p-value < 0.01), and 88 loci that are presumed to be under selection (each with a p-value < 0.05). An in-house python script using the Biopython package (Cock et al., 2009) was used to determine the longest open reading frame for each marker, which was then translated to amino acid sequences using the reference and alternative allele, respectively. The two amino acid sequences generated based on the two alleles at each locus were then compared for identification of missense and nonsense mutations.

## Results

The 400 SNP loci (Supplementary Table 1) were identified by aligning one CDS per locus to the reference genome, generating a maximum of two alleles at a bi-allelic locus. However, the SeqSNP based sequencing of the 288 genotypes targeting these SNP loci revealed three alleles at 30 loci and four alleles at three loci. Contrary to this, singletons (only the reference allele) were detected at 15 of the 400 loci across the 288 individuals resulting in 352 bi-allelic SNP loci. Hence, mono-, bi-, tri-, and tetra-allelic loci accounted for 3.8%, 88%, 7.5%, and 0.8% of the 400 loci targeted for genotyping of the 29 populations (288 individuals). Interestingly, no additional sequence variation was detected within 75 bp range up and downstream of the 400 target SNP loci during the original alignment of a single CDS to the reference genome. However, *de novo* SNP calling by mapping the reads from the 288 individuals to the reference genome led to the discovery of an additional 296 bi-allelic SNPs (Supplementary Table 1), clearly indicating the advantages of using the SeqSNP genotyping method. Due to the complexity of analyzing tri- and tetra-allelic loci, only the bi-allelic SNPs were included in the population genetic analyses of the 29 accessions. However, the tri- and tetra-allelic loci identified in this study can be further investigated, for example to determine the ploidy of individual red clover genotypes. Among the 352 bi-allelic SNP loci, 25 loci had missing values of over 5% and hence they were excluded. Overall, 623 SNPs that include 327 original SNPs and 296 *de novo* discovered SNPs were used for final data analysis (Supplementary Table 1). The translation of the coding sequences containing the bi-allelic SNPs revealed that 362 of the 623 SNPs induced missense mutations (data not shown).

### Population Structure, F-Statistics and Analysis of Molecular Variance

**FIGURE 1:**
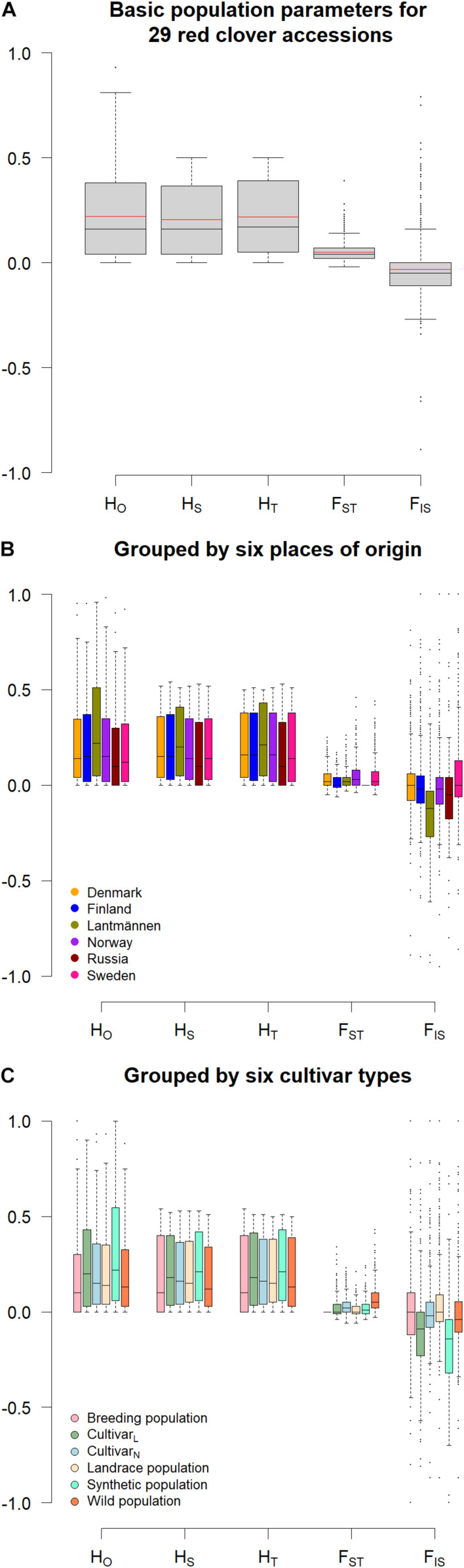
A boxplot visualizing the summary of different population genetics parameters for **(A)** all accessions, together with a red line marking the mean values, **(B)** each group of accessions grouped according to their origin, **(C)** each group of accessions grouped according to their type. The y-axis refers to the range of values for the parameters whereas the different parameters were given along the x-axis. H_*O*_ = observed heterozygosity; H_*S*_ = within population gene diversity; H_*T*_ = overall gene diversity; F_ST_ = population differentiation; F_IS_ = inbreeding coefficient.

This study revealed a low genetic distance between the accessions, where the mean Nei’s standard genetic distance of the accessions were quite similar, ranging only from 0.024 to 0.029 (Table 1). The inbreeding coefficient (F_IS_) of known diploids ranged from −0.05 to 0.03 whereas that of known tetraploids ranged from -0.33 to -0.29. Those with unknown ploidy had F_IS_ ranging from −0.08 to 0.06 (Table 1) spanning the ranges for the known diploids. The average observed heterozygosity (H_*O*_) across all loci and accessions was 0.22 with individual values ranging from 0.16 (accession NGB1009 and NGB24176) to 0.36 (accession SW RK1158; Table 1 and Figure 1A). The within population diversity (H_*S*_) ranged from 0.16 (accession NGB1009 and NGB24176) to 0.26 (SW RK1166) with an overall average of 0.21 (Table 1 and Figure 1A). The overall diversity (H_*T*_) mean estimate was 0.22 and share similar range of values with that of H_*S*_ (Figure 1A). The mean diversity estimate among the accessions (F_ST_) was 0.05, whereas the mean inbreeding coefficient (F_IS_) was −0.04 (Figure 1A). The summary of population genetics parameters by grouping the accessions according to their origin and type is presented in Figures 1B,C, respectively. Larger differences were observed between the groups in H_*O*,_ F_ST_ and F_IS_ as compared to H_*S*_ and H_*T*_ for both origin and type-based groupings of the accessions (Figures 1B,C). There was low variation between the groups in general; however, most of the variation was between accessions from Lantmännen and NordGen (Figures 1B,C). This is most prominently shown by the F_IS_ values. Interestingly, the within population variation (H_*S*_) of the single Russian accession (NGB24176) had the lowest mean value but the range of the individuals was corresponding to the other groups. The range of estimated values of population differentiation (F_ST_) was larger for accessions from Sweden and Norway compared with those from other sources (Figure 1B) and for wild types and Lantmännen cultivars (Figure 1C).

The AMOVA analysis on the 29 accessions (Table 2) prior to grouping revealed a highly significant (*P* < 0.001) differentiation among the accessions accounting for 5.4% of the total variation. AMOVA was then conducted by grouping the 29 accessions or their subsets into different groups according to origin, population type, maturity type and ploidy. In all cases, a highly significant differentiation was obtained among accessions within groups (*P* < 0.001) explaining from 0.5% to 1.8% of the total variation (Table 2). A low but significant differentiation (*P* = 0.012) was revealed when the eight Lantmännen accessions were compared with the 21 NordGen accessions, accounting for 0.5% of the total variation. Similarly, significant differentiation (*P* = 0.018) was obtained among groups of NordGen accessions from different countries, accounting for 0.9% of the total variation. The differentiation between different population types of the NordGen accessions was highly significant (*P* < 0.001) with 1.8% of the total variation residing among them. The eight Lantmännen accessions were grouped based on three different criteria (ploidy, maturity type and population type). A low but significant differentiation was obtained among diploids and tetraploids, accounting for 1.1% of the total variation (*P* = 0.02). However, no differentiation was observed between cultivars and synthetic populations as well as between different maturity groups. Even though there was significant structure distinguishing the accessions, the variation explained by each grouping criteria was very low. The highest variation explained by grouping the accessions was between the NordGen population types, accounting for 1.8 % of the total variation.

**TABLE 2:** Summary of analysis of molecular variance (AMOVA) based on 1000 permutations for (1) all 29 accessions without grouping, and by grouping (2) the 21 accessions from NordGen, and (3) the eight Lantmännen accessions.

Source of variation	Degrees of freedom	Sum of squares	Variance components	Percentage of variation	Fixation indices	Probability (*P*) value
Among accessions	28	3777.030	0.3563 V_a_	5.45	F_ST_ = 0.054	V_a_ and F_ST_ = 0.0000
Among individuals within Accessions	259	15579.36	−5.169 V_b_	–7.48	F_IS_ = −0.079	V_b_ and F_IS_ = 1.0000
Within individuals	288	20301.0	70.49 V_c_	102.03	F_IT_ = −0.02	V_c_ and F_IT_ = 0.9550
Total	575	39657.4	69.08			

^a^Among groups	1	214.51	0.356 V_a_	0.51	F_CT_ = 0.005	V_a_ and F_CT_ = 0.0117
Among accessions within groups	27	3562.51	3.341 V_b_	4.82	F_SC_ = 0.048	V_b_ and F_*SC*_ = 0.0000
Within accessions	547	35880.36	65.595 V_c_	94.66	F_ST_ = 0.053	V_*c*_ and F_ST_ = 0.0000
Total	575	39657.39	69.292			

^b^Among groups	4	726.833	0.63 V_a_	0.94	F_CT_ = 0.009	V_a_ and F_*CT*_ = 0.0185
Among accessions within groups	16	2119.99	3.55 V_*b*_	5.34	F_*SC*_ = 0.054	V_*b*_ and F_*SC*_ = 0.0000
Within accessions	395	24601.52	62.28 V_*c*_	93.71	F_ST_ = 0.063	V_*c*_ and F_ST_ = 0.0000
Total	415	27448.34	66.46			

^c^Among groups	3	717.65	1.186 V_a_	1.78	F_CT_ = 0.018	V_a_ and F_CT_ = 0.0000
Among accessions within groups	17	2129.17	3.182 V_b_	4.77	F_SC_ = 0.049	V_*b*_ and F_*SC*_ = 0.0000
Within accessions	395	2460.52	62.282 V_c_	93.45	F_ST_ = 0.065	V_*c*_ and F_ST_ = 0.0000
Total	415	27448.34	66.650			

^d^Among groups	1	158.31	0.818 V_a_	1.08	F_CT_ = 0.010	V_a_ and F_CT_ = 0.0244
Among accessions within groups	6	557.61	0.932 V_b_	1.23	F_SC_ = 0.012	V_*b*_ and F_SC_ = 0.0000
Within accessions	152	11293.45	74.30 V_c_	97.70	F_ST_ = 0.023	V_c_ and F_ST_ = 0.0000
Total	159	12009.40	76.05			

^e^Among groups	1	97.36	−0.076 V_a_	–0.10	F_CT_ = −0.001	V_a_ and F_CT_ = 0.5796
Among accessions within groups	6	618.59	1.440 V_b_	1.90	F_SC_ = 0.019	V_*b*_ and F_SC_ = 0.0000
Within accessions	152	11293.45	74.299 V_c_	98.20	F_ST_ = 0.018	V_*c*_ and F_ST_ = 0.0000
Total	159	12009.41	75.662			

^f^Among groups	1	128.12	0.377 V_a_	0.50	F_CT_ = 0.005	V_a_ and F_CT_ = 0.0880
Among accessions within groups	6	587.84	1.184 V_b_	1.56	F_SC_ = 0.016	V_*b*_ and F_*SC*_ = 0.0000
Within accessions	152	11293.45	74.299 V_c_	97.94	F_ST_ = 0.020	V_c_ and F_ST_ = 0.0000
Total	159	12009.41	75.859			

*^*a*^The 29 accessions grouped into two according to source: NordGen and Lantmännen.*

*^*b*^The 21 NordGen accessions grouped into five groups according to country of origin.*

*^*c*^The 21 NordGen accessions grouped into four population types: breeding populations, cultivars, landraces and wild.*

*^*d*^The eight Lantmännen accessions grouped into two ploidy groups: diploids and tetraploids.*

*^*e*^The eight Lantmännen accessions grouped into two population types: cultivars and synthetic populations.*

*^*f*^The eight Lantmännen accessions grouped into two maturity groups: early-middle late and late.*

**FIGURE 2:**
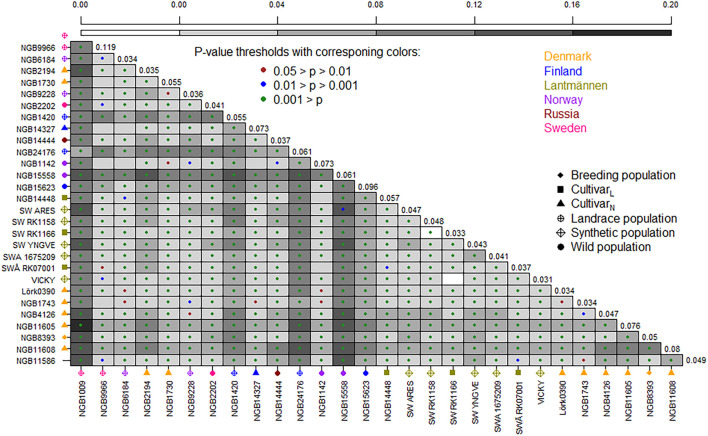
A graphical illustration of pairwise F_ST_ values between populations. The range of background color from light to dark corresponds to low to high F_ST_ values. The color of a dot in each square indicates the level of significance with brown for 0.05 > *p* > 0.01, blue for 0.01 > *p* > 0.001 and green for *p* < 0.001. The average F_ST_ values are given in the diagonal. The accessions are given on x- and y-axes, with corresponding label shape and color representing population type and country of origin, respectively (see figure keys). Cultivar_*L*_ = cultivar from Lantmännen; Cultivar_*N*_ = Cultivar from NordGen.

The F_ST_ based pairwise differentiation matrix between accessions is illustrated in Figure 2, where the darker color indicates higher differentiation between the accessions. As shown in this figure, seven accessions had highly significant (*P* < 0.001) pairwise differentiation with average F_ST_ values ranging from 0.07 to 0.11. These are Swedish (NGB1420 and NGB1009), Russian (NGB24176) and Norwegian (NBG15558 and NGB15623) wild accessions, as well as the Danish cultivars (NGB11605 and NGB11608), all of which were obtained from NordGen. The pairwise F_ST_ between two Lantmännen accessions, SW RK1166 (a synthetic population) and ‘Vicky’ (a cultivar) was 0, thus suggesting that they are closely related and might have been developed based on the same source. Additionally, the differentiation between SW RK1166 and SW RK1158 (synthetic populations from Lantmännen) was also 0, thereby suggesting that they too might have been developed from the same source. Interestingly, ‘Vicky’, SW RK1166 and SWRK1158 are three of the four known tetraploids included in this study. The fourth tetraploid accession (SWA 1675209) showed slightly higher differentiation from these three tetraploids.

**FIGURE 3:**
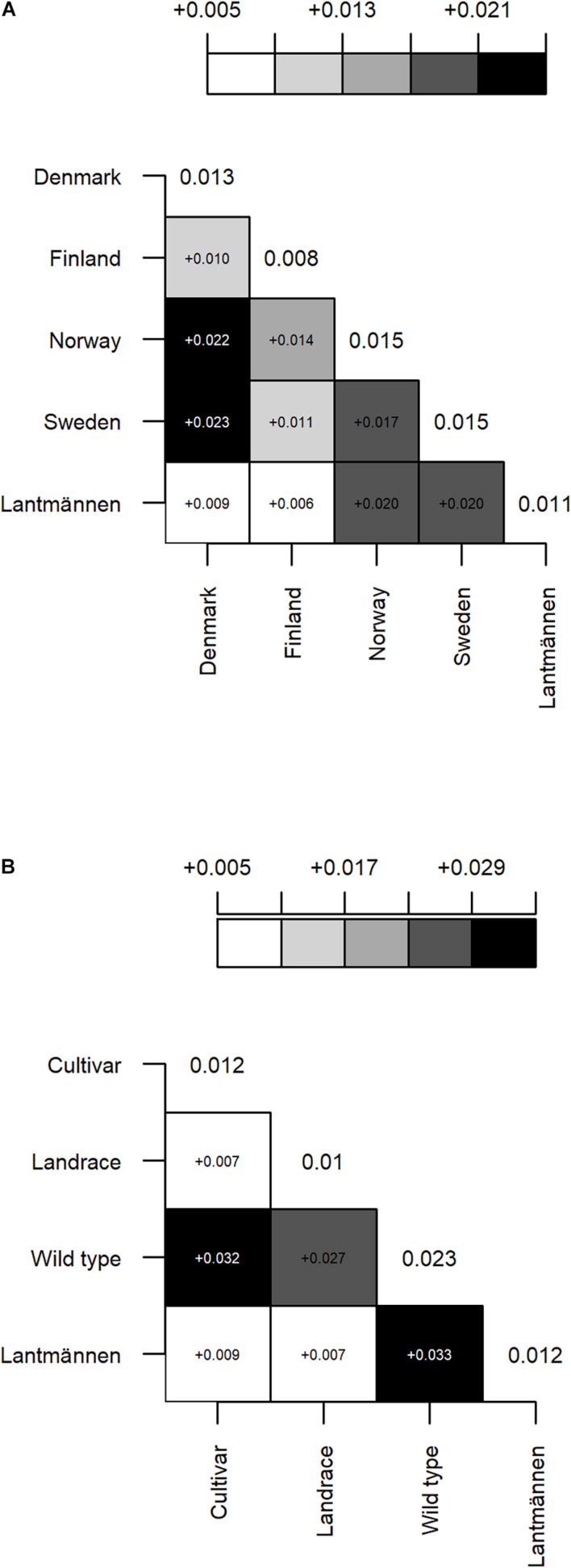
A graphical illustration of pairwise F_ST_ between groups of accessions representing different **(A)** country of origin: Sweden, Norway, Denmark, Finland and Lantmännen, and **(B)** population type: cultivar, landrace and wild populations from NordGen, and Lantmännen accessions. The gradient color intensity corresponds to low to high F_ST_ values (the deeper the color the higher the F_ST_ value). All values are significant at a threshold value of 0.05. F_ST_ value of each pair is shown in the corresponding square. The diagonal values are mean F_ST_ values of each group. Lantmännen is represented as a separate group under country of origin.

A matrix illustrating the pairwise F_ST_ values between groups of accessions according to their origin (Figure 3A) shows a low differentiation between accessions from Lantmännen and those from Denmark or Finland (F_ST_ = 0.009 and 0.006, respectively). The differentiation between Finland and Denmark was also low (F_ST_ = 0.010), which is interestingly half the value of the differentiation between Denmark and Norway or Sweden (F_*ST*_ = 0.022 and 0.023, respectively). Furthermore, the differentiation between Denmark and Sweden or Norway are almost equal to the differentiation between Lantmännen and Sweden or Norway (F_ST_ = 0.020 for both; Figure 3A). The pairwise F_ST_ values between population types (Figure 3B) showed similarly low differentiation of NordGen cultivars and landrace populations from Lantmännen accessions, whereas the NordGen wild populations showed a relatively higher differentiation from the Lantmännen accessions (F_ST_ = 0.033). Overall, the wild populations are the most differentiated among these groups with average F_ST_ value of 0.023 (Figure 3B).

### Cluster Analysis and Discriminant Analysis of Principal Components

**FIGURE 4:**
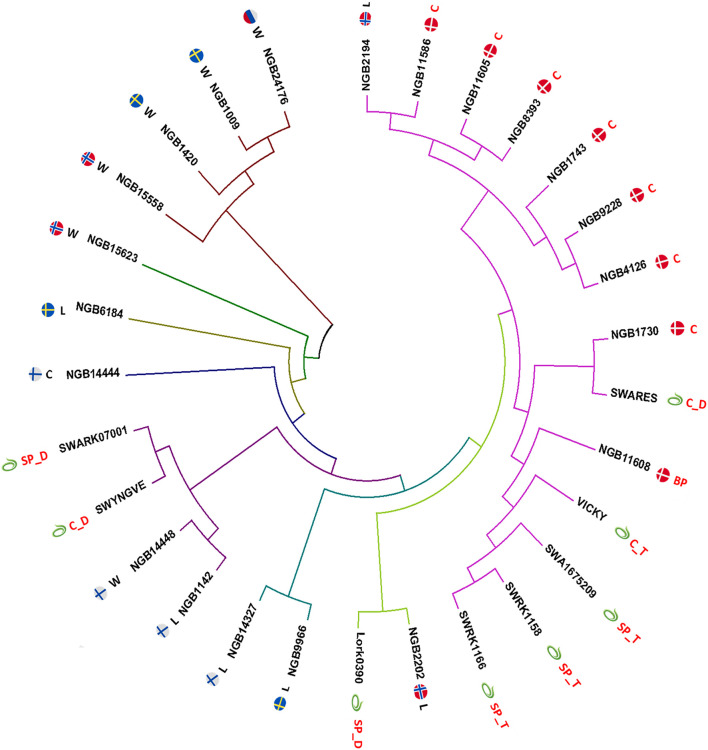
A neighbor joining (NJ) tree constructed based on Nei’s unbiased genetic distance values of the 29 accessions with a fan layout and edges scaled to equal length. Each node is labeled with the accession name as well as an icon of the flag of its country of origin or the Lantmännen symbol. The letter abbreviations following the flag corresponds to the population type of the accession with W for wild population, L for landrace, C for cultivar, SP for synthetic population and BP for breeding population. The letter following the underscore (_) signifies the ploidy of the accession (if it is known) with D for diploid and T for tetraploid.

A neighbor joining (NJ) tree (Saitou and Nei, 1987) constructed based on the Nei’s unbiased genetic distance between each pair of the 29 accessions revealed small and large clusters as well as solitary accessions (Figure 4). Five of the six wild accessions were clearly separated from the cultivated groups with four of them forming a separate cluster despite representing different countries (Norway, Russia and Sweden). However, a wild accession from Finland (NGB14448) was clustered with a landrace accession from Finland (NGB1142) and two Lantmännen diploid accessions (SW YNGV and SWÅ RK0700). The NJ tree also clearly depicted a close clustering of the Lantmännen tetraploid accessions while the four diploids were spread across three clusters. With regard to country of origin, the Danish accessions (which are all cultivars except one) were closely clustered together with the exception that two accessions (a cultivar and a breeding population) were placed in a separate but closely related cluster. Interestingly, the Lantmännen tetraploid accessions showed higher genetic similarity with the Danish accessions than with those from other countries, and were closely clustered with the NGB11608 (a breeding population). The diversity among the landrace accessions was also displayed in the NJ tree, where even those from the same country were placed far apart in different clusters.

**FIGURE 5:**
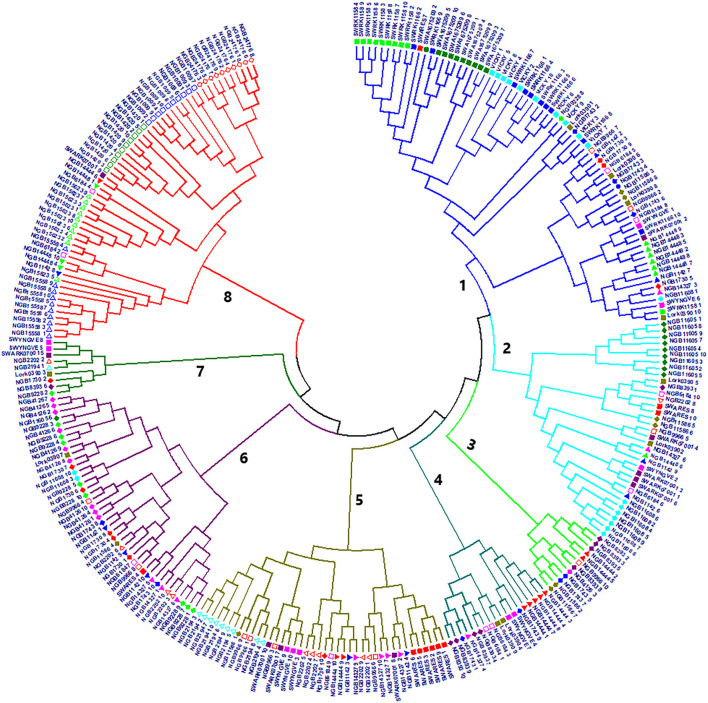
A neighbor joining (NJ) tree of 288 individuals representing 29 accessions based on evolutionary distances computed by the Tamura-Nei method. Individuals sharing the same symbol and color belong to the same accession.

The genetic distance between the 288 individual genotypes calculated based on the Tamura-Nei method was used for NJ cluster analysis and resulted in eight clusters (Figure 5). The tree exhibited clear differences between the accessions in terms of the within accession genetic diversity. Interestingly, all genotypes of the four known tetraploid accessions (SW RK1158, SW RK1166, SWA 1675209 and ‘Vicky’) were clustered in cluster-1 whereas the genotypes of the four known diploids (LÖRK0390, SWÅ RK07001, SW ARES and SW YNGVE) were spread across three to five clusters. With the exception of five accessions representing wild populations, all NordGen accessions had members in more than one cluster. All genotypes of the NordGen wild accessions (NGB15558 and NGB15623 from Norway; NGB24176 from Russia; and NGB1009 and NGB1420 from Sweden) were clustered in cluster-8 with the exception of accession NGB14448 (from Finland) that had members in clusters-1, -6 and -8 (Figure 5).

**FIGURE 6:**
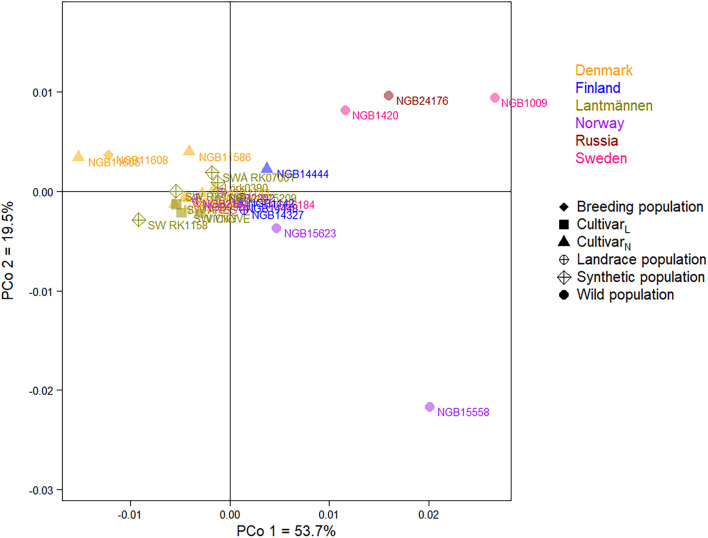
A principal coordinate analysis (PCoA) plot depicting the relationship between the 29 red clover accessions. Accessions with the same font-color belong to the same origin (a country or Lantmännen; see Figure key). Accessions with the same label-shape and color belong to the same population type (see Figure key).

A Nei’s unbiased genetic distance based two-dimensional PCoA plot depicting the relationship between the 29 accessions (Figure 6) explained 53.7% and 19.5% of the total variation in the first and second principal coordinates, respectively. The PCoA showed a close relationship among the majority of the accessions forming a major cluster. However, the wild populations were clearly separated from the rest with the exception of the Norwegian wild population NGB15623 that was placed close to the major cluster. Interestingly, the two Swedish (NGB1009 and NGB1420) and the Russian (NGB24176) wild populations were placed close to each other while the Norwegian wild population (NGB15558) was separated from them along the second principal coordinate. Among the seven Danish accessions, two cultivars (NGB11586 and NGB11605) and a breeding population (NGB11608) were slightly separated from the main cluster along with a Finnish cultivar (NGB14444).

**FIGURE 7:**
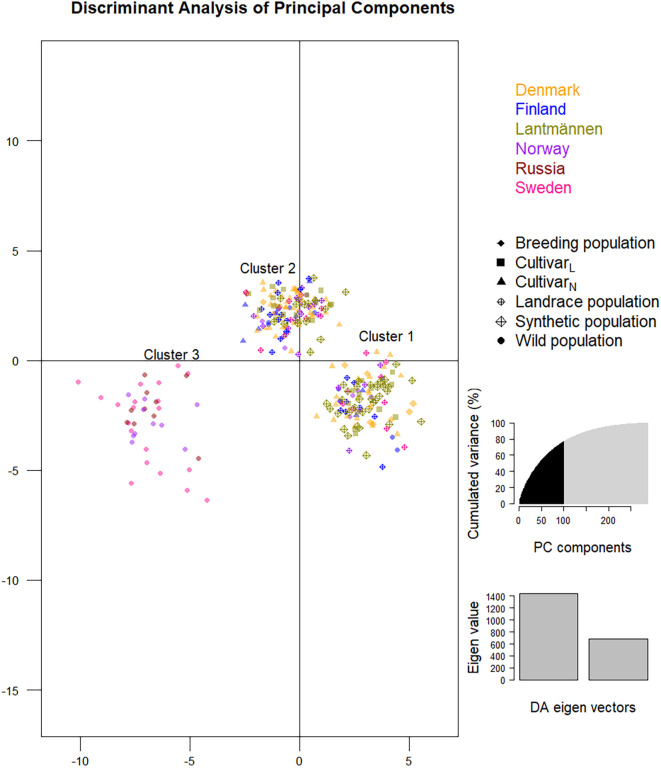
A discriminant analysis of principal components (DAPC) depicting the clustering of the 288 red clover genotypes of the 29 accessions into three clusters. Genotypes sharing the same label-color belong to the same origin (a country or Lantmännen; see Figure key) whereas genotypes sharing the same label-shape belong to the same population type (see Figure key). The graph for the selection of 100 principal components and Eigenvalues of the discriminant analysis are shown on the right.

The DAPC analysis (Figure 7) accounted for 76.9% of the cumulative variance and separated the 288 individuals into three clusters (the cluster allocation of members of each accession is given in Table 1). All individual genotypes of four accessions representing wild populations from Sweden (NGB1009 and NGB1420), Norway (NGB15558) and Russia (NGB24176) clearly separates from the rest and formed Cluster-3. These accessions also had the highest mean F_ST_ values (diagonal values in Figure 2) and were clearly separated from the main PCoA cluster (Figure 5). The remaining populations were split over cluster-1 and cluster-2.

### Loci Deviated From Hardy-Weinberg Equilibrium and Loci Under Selection, and Their Corresponding Mutation Types

The HWE test revealed that 51 out of the 623 SNP loci, showed a highly significant deviation (*P* < 0.01) from HWE. Eighteen of these loci showed excess heterozygosity whereas 33 loci were heterozygote deficient (Supplementary Table 2). The translation of the coding sequences containing the 51 SNP loci using their alternative alleles resulted in missense mutations in 28 loci that led to amino acid changes and 1 nonsense mutation that resulted in premature termination of the amino acid sequence (Supplementary Table 2). The nonsense mutation is located in the oxysterol binding protein (OSBP)-related protein 1C gene (The UniProt Consortium, 2021), which is involved in the regulation of sterol transportation. The changes in the remaining 22 loci were same-sense mutations.

The examination of the joint distribution of F_ST_ and the heterozygosity at the 623 loci under a non-hierarchical finite island model resulted in 88 loci that had significant F_ST_ P-values (<0.05), and were therefore considered to be under selection. The translation of the coding sequences of genes containing these loci using the alternative SNPs resulted in 42 and two missense and nonsense mutations, respectively (Supplementary Table 3). The remaining 44 SNPs did not lead to change in amino acids.

**FIGURE 8:**
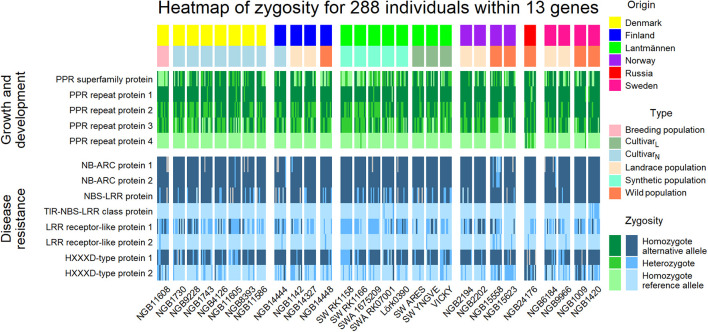
A heatmap illustrating the zygosity level of five PPR proteins, known to play an important role in growth and development, and eight disease resistance proteins in the 288 individuals. The individuals were grouped according to their accession and the top annotation sorted the individuals further into place of origin and type. Gray fields indicate missing genotype for that individual at the particular locus.

All the alternative allele substitutions that were shown to be under selection and were located within genes coding for PPR proteins resulted in missense mutations. The assessment of the zygosity of the 288 individuals revealed differences in the level of heterozygosity for the PPR proteins between the wild populations and Lantmännen cultivars (Figure 8). Among the PPR proteins, the PPR repeat protein 1 marker differs a lot within the 29 accessions but the reference allele seems to be preferred within the Danish breeding population and the Lantmännen synthetic populations. Furthermore, homozygosity of the reference allele was absent for PPR repeat protein marker 2. For PPR repeat protein marker 4, all individuals except two synthetic accessions and five of the Russian wild type individuals are homozygous for the reference allele. The overall level of heterozygosity in the PPR proteins are higher in the four tetraploid accessions comprising three synthetic populations (SW RK1158, SW RK1166, and SWA 1675209) and a cultivar (‘Vicky’) from Lantmännen.

Within the group of disease resistance proteins, one of the mutations within the HXXD-type acyl-transferase family protein (HXXD-type protein 1 in Figure 8) was a missense mutation (Supplementary Tables 1, 2 and Figure 8). For most of the loci within disease resistance genes, one of the two alleles were more dominant, and hence the level of heterozygosity is lower and individual genotypes are mostly homozygous for either the reference or alternative allele. The LRR receptor-like protein 1 marker is the most heterozygous loci of the eight. Interestingly, the alternative alleles at the HXXP protein 1 locus resulted in premature termination of the amino acid sequence, and the analysis using the Protein Variation Effect Analyzer (PROVEAN; Choi and Chan, 2015) revealed that this mutation has a deleterious effect on the protein. For this locus, homozygous individuals for alternative allele were preferred (Figure 8). Thus, the mutation to alterative alleles in these loci might have noteworthy effects on the genotypes’ disease resistance and consequently survival.

## Discussion

In the present study, SeqSNP is used as genotyping method for genotyping the target SNP loci. The method also allows *de novo* discovery of new SNPs near the target SNP loci, which resembles genotyping by sequencing (GBS). However, it should be noted that our approach might have excluded the possibility of discovering SNPs in previously uncharacterized genes of potential significance in red clover improvement. The 623 bi-allelic polymorphic markers genotyped across 288 individuals representing the 29 red clover accessions using SeqSNP revealed significant population structure differentiating accessions due to origin and cultivar type. Four wild accessions, two Swedish (NGB1420 and NGB1009), one Russian (NGB24176) and one Norwegian (NGB15558) were consistently grouped together while separated from the other accessions. Additionally, the known tetraploids (‘Vicky’, SWA 1675209, SW RK1166, and SW RK 1158) grouped together in the NJ trees while the known diploids were dispersed across different clusters together with NordGen accessions. Furthermore, the tetraploids showed similar patterns in zygosity in markers associated with growth and development (Figure 8).

### F-Statistics, Heterozygosity and Within Population Diversity

The fixation indices measure inbreeding for each locus (Wright, 1949, 1965) and a maximum value of one is attained when all individuals in an accession are homozygous at a locus. Negative values of F_IS_ indicate excess heterozygosity. In the present study, The F_IS_ values ranged from −0.33 to 0.06 with 45% of the accessions having negative values. The mean F_IS_ value (−0.31) of the tetraploids were significantly lower than that of the diploids (−0.01) as expected, because there is higher probability of heterozygosity in tetraploids than in diploids under an outcrossing reproductive system. The F_IS_ values for the known diploids ranged from 0.05 to 0.03 whereas that of the 21 NordGen accessions with unknown ploidy ranged from 0.08 to 0.06 (mean = 0.00). Jones et al. (2020) also reported similar F_IS_ values for red clover ecotypes (diploids) from United Kingdom in their GBS based study. Whereas the ecotypes from central Europe recorded a slightly higher F_IS_, suggesting the Scandinavian red clover may be more similar to that of United Kingdom than to those of central Europe. In a recent bi-allelic SNP marker-based study by Tsehay et al. (2020) in *Guizotia abyssinica* (a strictly outcrossing diploid species; Geleta and Bryngelsson, 2010), a mean F_IS_ value of 0.13 was reported, which is higher than the values obtained in red clover. This could suggest that red clover is as strictly outcrossing as *G. abyssinica* if not stricter.

The mean values for observed heterozygosity (H_*O*_) for tetraploids (0.35) was higher than that of the diploids (0.21) and accessions with unknown ploidy (0.20). The average observed heterozygosity for European red clover ecotypes in Jones et al. (2020) was 0.25. The F_IS_ and H_*O*_ values suggest that all 21 accessions with unknown ploidy obtained from NordGen are most likely diploids. Overall, the heterozygosity and F_IS_ values obtained in this study (Figure 1A) are in line with expected values in an outcrossing species (Schoen and Brown, 1991; Huang et al., 2019). Similar to observed heterozygosity, the within accession diversity was higher for the tetraploids (mean H_*S*_ = 0.25) when compared to all other accessions (mean H_*S*_ = 0.20). According to Öhberg (2008) and Vleugels et al. (2013) tetraploid red clover were shown to have higher persistence than diploid red clover. Furthermore, Ergon et al. (2019) indicated that there were differences in allele frequencies in red clover survivor populations after exposure to certain environmental stresses vis-á-vis the original populations. Generally, they showed that higher genetic diversity within populations resulted in higher chance of survivors following exposure to various selection pressures. Thus, the higher within accession diversity shown by the present study in tetraploids could be a result of persistence and resistance breeding by Lantmännen. Persistence and resistance are two of the major differentiating traits between tetraploid and diploid red clover in an agricultural perspective together with seed yield and dry biomass. The tetraploid red clover produces less seeds due to either a lower number of flower heads or higher levels of embryo abortion compared to diploids (Vleugels et al., 2015). The low seed production is a disadvantage for seed producers but the tetraploid red clover is more competitive for the traits regarding quality fodder such as dry biomass yield, persistence and resistance (Taylor and Quesenberry, 1996b).

It is interesting that the wild accessions had lower average within population diversity than the other groups with four of the six accessions recording the lowest H_*S*_ values (Table 1). This is likely due to the dominance of wild type alleles over mutant alleles in the wild populations (with mutant alleles having lower frequencies than in the cultivated accessions). This is in line with the generally lower percent polymorphic loci (%PL) obtained in the wild accessions compared to the cultivated ones. The overall mean H_*S*_ and H_*T*_ (0.21 and 0.22, respectively) in the present study was slightly lower than those reported in Jones et al. (2020) suggesting a slightly lower genetic diversity in Scandinavian red clover as compared to the Central European ones. However, the differences could also be due to differences in the number of markers used and the genomic regions targeted. In the present study, gene-coding regions were targeted whereas the GBS based study in Jones et al. (2020) was not specifically targeting genes and hence higher genetic variation is likely. The comparison of different population genetics parameters between origin and type-based groups of accessions showed that the difference is very low in general, but it was relatively higher between the NordGen accessions and Lantmännen, which was clearly due to the presence of tetraploids in the latter.

The range of F_ST_ values was larger for accessions from Sweden and Norway (Figure 1B) as well as for both wild populations and Lantmännen cultivars (Figure 1C). The results agree with the pairwise F_ST_ values for accessions grouped according to origin and cultivar type (Figures 3A,B). Sweden and Norway accounted for two-thirds of the wild accessions included in the present study. Presumably, the genetic variance between the wild populations had stronger contribution to the wider range in F_ST_ values obtained for accessions from these two countries, due to restricted gene flow in nature. On the other hand, the wider range in F_ST_ values observed within the Lantmännen group was contributed by the presence of both diploids and tetraploids. There were relatively higher pairwise F_ST_ values between Russia, Sweden and Norway (Figure 3A) but low values between the Lantmännen, Finnish and Danish accessions (Figure 3B). The accessions representing Denmark are all cultivars except a single breeding population, and most of the Finnish material represents cultivated gene pool, which can explain the lower F_ST_ values.

The Norwegian and Swedish populations, on the other hand, included only wild and landrace accessions explaining the higher F_ST_ values. The effects of wild populations versus cultivars are further noted in Figure 3C where the F_ST_ values are lower between the cultivated types and higher between cultivars and wild populations. Furthermore, it is worth noting that the landraces are closer to the cultivars than to the wild populations. Landraces are cultivated types whose genetic diversity is shaped through traditional methods of selection and not through modern breeding programs. It can be hypothesized that different landraces have been developed independently and selection by human has been less stringent (than the case in cultivars), and thus contain a higher level of within population genetic diversity and have higher F_ST_ values compared to the modern cultivated types that include breeding and synthetic populations and cultivars. However, the present study revealed that genetic diversity and population differentiation of all cultivated types are generally similar indicating that modern red clover breeding programs did not lead to loss of genetic diversity and affect population structure. As suggested by Jones et al. (2020) the relatively short period in the cultivation and breeding of red clover could have contributed to similar level of genetic diversity and low differentiation between its wild and cultivated gene pools.

### Cluster Analysis, Principal Coordinate Analysis and Discriminant Analysis of Principal Components

As previously discussed, red clover is a strictly outcrossing species, and outcrossing species have a greater gene flow than self-pollinating species due to the role of their pollinators. This increased gene flow between subpopulations reduces the difference between them. From the AMOVA (Table 2), 5.4% of the total variation was explained by the variation among accessions, which is slightly higher than that of a strictly outcrossing species *G. abyssinica* (Tsehay et al., 2020). Compared with studies on *Arabidopsis thaliana* (Brennan et al., 2014) and both outcrossing and selfing *Zingiber* (Huang et al., 2019), which generated 81.6%, 65.3% and 91.7% variation among accessions, respectively, the differentiation between red clover accessions are in the lower range. In these examples, the difference between the three outcrossing species is interesting with 65.3% for the outcrossing *Zingiber*, 5.4% for red clover and 4.5% for *G. abyssinica*. *Zingiber* is less strictly outcrossing than red clover or *G. abyssinica*, which can be a reason for the great difference in variation explained among accessions. This research finding suggests that the degree of stringency of the outcrossing reproductive system can be shown through the AMOVA results. However, there are other contributors to the variation between accessions other than the breeding system. One factor that should not be over looked is the effect of sample selection. The low level of population structure presented in this study is in line with the results of Dias et al. (2008), who reported that the vast majority of the variance in red clover were resided within the populations although the level of differentiation was relatively higher in their study. Similarly, Jones et al. (2020) reported a significant but low population differentiation (F_ST_ = 0.076) in their GBS based study that included European and Asian ecotypes and cultivars.

AMOVA analysis was further performed after six separate criteria were applied to group the 29 accessions. Four of the groupings revealed a very low but significant differentiation between the groups. Overall, the variation explained by the groupings was very low with only NordGen accessions grouped by population types and the Lantmännen accessions grouped by ploidy explaining over 1% of the total variance (1.78% and 1.08%, respectively).

The cluster analysis at both accession and individual genotype levels demonstrated the genetic relationship between the accessions used in the present study. The eight Lantmännen accessions displayed interesting pattern at both levels. The four tetraploid accessions are closely related and all accessions and their individual members were placed in the same cluster. The result suggests a narrow genetic base of the tetraploid red clover and hence crossbreeding among them may not lead to a significant improvement of desirable traits. Since tetraploid red clover is a result of successful plant breeding, based on limited tetraploid genotypes developed through chromosome doubling (Sjödin and Ellerström, 1986; Taylor and Quesenberry, 1996b), the obtained results are not unexpected. Hence, further genetic analyses among a larger set of tetraploid accessions need to be conducted to identify genetically distinct groups suitable for crossbreeding. On the other hand, the Lantmännen diploid accessions are diverse and appeared to have close relationship with landraces or cultivars from Finland, Norway, Sweden and Denmark. This indicates that the diploid cultivars and synthetic populations that are currently in use at Lantmännen largely represents the landrace gene pool from the different countries in Scandinavia.

Similar to the cluster analyses, the relatively close relationship between the cultivated accessions was also shown in the PCoA, where they were clustered close to the center of the PCoA bi-plot with a slight separation of the three Danish cultivars (NGB11586, NGB11708, and NGB11605) and one Finnish cultivar (NGB14444). The relatively higher divergence between the wild accessions as compared to the case of cultivated pool is expected due to a limited gene flow among the wild populations. In agreement with the cluster analyses, five of the six wild accessions were separated from the other groups along the principal coordinate 1, which accounted for 54% of the total variation whereas the differentiation between them is clearly observable along the principal coordinate 2 (Figure 6). The clear separation of most wild accessions from the cultivated gene pool is mainly the results of human selection, and is in agreement with the results of previous research (Shim and Jørgensen, 2000; Geleta et al., 2007). The case of the Finnish wild accession NGB14448 is interesting. Unlike all other wild accessions, it was closely grouped with cultivated accessions including a Lantmännen cultivar (at accession level), as shown by cluster analysis, PCoA and DAPC. At individual level, its genotypes were placed in different clusters, of which three individuals clustered with the other wild genotypes. This may suggest that the accession is a result of mixture of seeds (accidental or intentional) from cultivated and wild accessions. Hence, it is interesting to investigate this accession further and other similar accessions at NordGen.

Unlike the PCoA bi-plot that was the result of the first two principal coordinates, the DAPC, which described 100 principal components (Figure 7), led to three significant clusters (Table 1). It shall be noted that the PCoA was analyzed based on Nei’s unbiased genetic distance while the principal components of DAPC was based on an Euclidian distance. Hence, the depiction of the PCoA is a representation of the variation between accessions on a more genetic level while Euclidian distance is strictly mathematical explanation of the variation between SNP genotypes. The separation of the first two DAPC clusters is not clear-cut since they are a mixture of different population types and from different origins. The lack of complete differentiation between cluster one and two with regard to population affiliation can be explained by a high level of within population variation (Figure 1A). One exception is the known tetraploid SW RK11158 for which all its individuals were located in cluster-2. Interestingly, the other tetraploids also had the majority of their individuals in cluster-2. The third DAPC cluster contained all individuals of four of the six wild populations, NGB1009 (Sweden), NGB1420 (Sweden), NGB15558 (Norway) and NGB24176 (Russia), which were also clustered together in the NJ trees and PCoA bi-plot. The affiliation of populations in the DAPC clusters are further confirmed by the results of the individual NJ tree (Figure 5), which showed a similar clustering pattern.

### The Differentiation Between Accessions Shown by Markers of Interest

The 623 SNP loci used in the present study are located within the coding sequences of 231 genes, of which 51 loci (8.2%) showed significant deviation from HWE. Because, red clover is an outcrossing species with a gametophytic self-incompatibility system (Taylor, 1982), excess heterozygosity is likely even at neutral loci. However, excess heterozygosity coupled with nonsense mutation, such as the case at locus ASHM01029522C (Supplementary Table 2), within the oxysterol binding protein (OSBP)-related protein 1C gene (The UniProt Consortium, 2021) regulating the sterol transportation, could be due to heterozygote advantage. On the other hand, heterozygote deficiency in plants with a self-incompatibility system, strongly suggests that the loci may have fitness values in the form of homozygote advantage or linked to such loci. Two-thirds of the 51 loci were heterozygote deficient, and further investigation of their potential roles in red clover fitness is therefore recommended. Li et al. (2019) reported that 28% of the SNPs identified using publicly available red clover RNAseq data accounted for missense mutations. As a comparison, the present study found that 58% of the SNPs gave rise to missense mutations. Among the SNPs shown to be under selection pressure, 48% resulted in missense mutations whereas missense mutations accounted for 55% of the loci deviated from HWE. There are multiple reasons for the differences in the proportion of missense mutations between the studies, which include differences in sequencing methods or the stringency of steps to determine missense mutations. The present study used DNA sequences of known coding regions at specific loci across 29 accessions while Li et al. (2019) used a combination of targeted genomic amplicon sequences of 72 genotypes of a breeding population and RNA-seq data from three genotypes of different cultivars. Furthermore, the present study used the longest open reading frame on each protein sequence with no additional penalties or weights applied in scoring. However, Li et al. (2019) used a software developed to score all types of mutations and might have been more stringent in calling missense mutations.

Among the 231 protein coding genes containing the 623 bi-allelic SNPs, those coding for the pentatricopeptide repeat (PPR) family proteins and proteins related to disease resistance are among the most frequent groups. The PPR protein family is one of the largest protein families in land plants and are known to regulate RNA expression and have an effect on respiration, photosynthesis, growth and development, and responses to environmental stresses (Barkan and Small, 2014). The zygosity pattern of markers located within the five PPR proteins and eight disease resistance proteins showed that there was a difference in heterozygosity between the Lantmännen and NordGen accessions. This is in agreement with the levels of H_*O*_ (Figure 1C) where Lantmännen populations were shown to have a higher level of heterozygosity.

The level of heterozygosity was higher in the PPR protein markers than in the disease resistance proteins and the SNP loci in the disease resistance genes were more often homozygous. Interestingly, the nonsense mutation (HXXXD-type protein 1 in Figure 8) showed that the majority of individuals were homozygous for the alternative allele, which was higher than expected based on the allele frequency. Using the prediction tool on protein function, PROVEAN (Choi and Chan, 2015), the premature stop codon induced by the alternative allele had significant effect on the protein function. It would therefore be interesting to further study the effect of the end terminal of the HXXXD-type protein and the response to pathogens. However, the result of selection based on HWE and F_ST_ could be due to association with another loci; an effect referred to as “hitch-hiking” by Smith and Haigh (1974). If there is linkage disequilibrium between one neutral locus and another locus under selection, the neutral allele will be inherited with the allele under selection thus appears to be under selection as per HWE an F_ST_ assumptions. De Vega et al. (2015) found, however, a low level of linkage disequilibrium between red clover markers, thus decreasing the possibility of a hitch-hiking locus.

## Conclusion

This research revealed the population structure of red clover accessions gathered from the Nordic countries based on SNPs chosen to represent variation in genes that probably regulate traits important in agriculture. The results show a significant differentiation among accessions from different countries as well as population types, even though it represented a small proportion of the total genetic variation. The inbreeding coefficient was significantly lower whereas observed heterozygosity and within population diversity were significantly higher in tetraploids than in diploids. This is in line with general expectation in a species with outcrossing reproductive system. The present study revealed high genetic similarity among the tetraploid accessions, suggesting a narrow genetic basis of tetraploid red clover being used at Lantmännen. A comprehensive genetic study, incorporating all available tetraploid accessions at Lantmännen and elsewhere, would reveal the overall genetic diversity of tetraploid red clover, which facilitates an adequate comparison with the diploids, as well as designing effective breeding programs. The comparative population genetics analysis of the tetraploids and diploids suggests that the NordGen red clover accessions included in the present study are diploids, as expected based on the background information. The comparison of the wild populations (ecotypes) with the cultivated ones showed a relatively lower within population diversity in the wild populations reflecting differences in allele frequency between the two groups, likely due to the dominance of wild type alleles across loci in the ecotypes. Most, but not all, wild red clover populations were distinctly separated from the cultivated gene pool. In relation to this, the case of wild accession NGB14448 that had highly similar genetic profile with that of some cultivated accessions needs further investigation, to rule out any misclassification at NordGen and to shed more light on the overall genetic relationship between the wild and cultivated red clover gene pool. Interestingly, all cultivated diploid accessions had similar levels of genetic diversity. Hence, modern red clover breeding programs did not lead to significant loss of genetic diversity. This study demonstrates how the breeding material used today reflects the red clover genetic diversity in the Nordic countries and provides the breeders with the knowledge needed to develop genomic breeding tools for red clover. Future research is needed using a greater number of both accessions and individuals together with phenotypic data to obtain a definitive conclusion about the genetic diversity in Nordic red clover and beyond.

## Data Availability Statement

The datasets presented in this study can be found in online repositories. The names of the repository/repositories and accession number(s) can be found in the article/Supplementary Material. All raw sequences are available at SRA, BioProject PRJNA765476.

## Author Contributions

MG secured the funding with RO and CH as co-applicants. JO and MG selected and ordered the seed material from NordGen and Lantmännen Seed, analyzed the data, and compiled the results with input from RO and CH. JO was responsible for the planting and taking care of the plant material in the greenhouse, together with MG and CH sampled the plant material, performed the SNP mining with the assistance of MG, and wrote the manuscript. All authors contributed to the design of the study, provided inputs, revised the manuscript, and approved its final version.

## Conflict of Interest

The authors declare that the research was conducted in the absence of any commercial or financial relationships that could be construed as a potential conflict of interest.

## Publisher’s Note

All claims expressed in this article are solely those of the authors and do not necessarily represent those of their affiliated organizations, or those of the publisher, the editors and the reviewers. Any product that may be evaluated in this article, or claim that may be made by its manufacturer, is not guaranteed or endorsed by the publisher.

## Footnotes

1
https://www.ncbi.nlm.nih.gov/assembly/GCA_900292005.1/

2
https://legumeinfo.org/

3
https://www.nordic-baltic-genebanks.org/gringlobal/search.aspx

4
https://www.lantmannenseed.com/

5
https://biosearchassets.blob.core.windows.net/assetsv6/guide_bioark-leaf-collection-kit.pdf

6
https://biosearch-cdn.azureedge.net/assetsv6/sbeadex-plant-data-sheet.pdf

7
https://www.uniprot.org/
